# A slow decay to extinction: cold temperatures and reduced microbial complexity prolong the persistence of planktonic *Francisella tularensis*

**DOI:** 10.1128/aem.01261-26

**Published:** 2026-09-29

**Authors:** Johanna Thelaus, Dawn Birdsell, Stina Bäckman, Malin Granberg, Emelie Salomonsson, Igor Golovliov, Eva Lundmark, David Sundell, Jonas Näslund, Laila Noppa, Anna-Lena Johansson, Karin Wallgren, Joseph D. Busch, Jason W. Sahl, Anders Johansson, Mats Forsman, David M. Wagner

**Affiliations:** 1 Division of CBRN Defence and Security, Swedish Defence Research Agency 201284, Umeå, Sweden; 2 Pathogen and Microbiome Institute, Northern Arizona University, Flagstaff, Arizona, USA; 3 Department of Clinical Microbiology and the Laboratory for Molecular Infection Medicine Sweden, Umeå University 8075, Umeå, Sweden; Universite de la Reunion, Ste Clotilde, France

**Keywords:** *Francisella tularensis*, persistence, water, planktonic, culturability, virulence, disease

## Abstract

*Francisella tularensis* is a zoonotic pathogen transmitted to humans through contact with infected animals, arthropod vectors, or contaminated environmental sources. Although replication occurs primarily within eukaryotic host cells, persistence outside a host is thought to be critical for long-term maintenance and transmission in natural ecosystems. To better understand persistence in aquatic habitats outside of mammalian hosts, we studied virulent strains of *F. tularensis* subspecies *tularensis* (type A) and subspecies *holarctica* (type B) incubated in a planktonic state (free living in water) under various axenic aquatic conditions. After 1 year in cold, nutrient-poor treatments, the type B strain (FSC200) caused disease in mice and remained culturable on laboratory media, whereas the type A strain (Schu S4) caused disease but with delayed onset and became non-responsive to laboratory growth conditions. In 1.5-year persistence experiments, type B still caused disease in mice, but with delayed onset, whereas type A no longer caused disease. The rate of bacterial decline depended on subspecies, temperature, and the initial inoculum size. We further explored the persistence of type A and type B populations in natural pond water, which corroborated findings from axenic conditions and revealed that both strains perpetuated disease from a non-replicative inactive persistent state. This study provides timeframes for the persistence of virulent planktonic *F. tularensis* subspecies *tularensis* and *holarctica* populations in various aquatic conditions, contributing to increased understanding of host-independent survival as a component of the perpetuation of tularemia.

**IMPORTANCE:**

Long-term persistence of planktonic (free living in water) *Francisella tularensis* subspecies tularensis (type A) and subspecies *holarctica* (type B) requires low temperatures and reduced microbial complexity. These conditions are typical of water wells, supporting the epidemiological observations that drinking water reservoirs may serve as sources of human *F. tularensis* infections (specifically type B). Importantly, persistence of types A and B did not correlate with molecular viability assays and culturability. This suggests that risk assessment of contagious bacteria based on these methods may not be appropriate. This is the first study to perform side-by-side comparisons of fully virulent strains of type A (Schu S4) and type B (FSC200), to evaluate persistence (defined by the capability to cause tularemia in mice) outside the host-restricted niche under stress conditions in water. The results contribute to the understanding of long-term persistence of *F. tularensis* between tularemia outbreaks in humans and other mammals.

## INTRODUCTION

The genus *Francisella* is composed of gram-negative, non-motile, non-spore-forming, aerobic bacteria with varied life cycles. Representatives of this genus inhabit a variety of ecological niches, many associated with aquatic environments (1). The zoonotic pathogen *Francisella tularensis*, etiological agent of tularemia and a host-associated facultative intracellular pathogen, likely evolved from a host-independent ancestor (1–4), but it now can only replicate inside or in close association with eukaryotic host cells (5), including human cells (6).

Multiple characteristics of *F. tularensis* led several countries to develop it as a potential biological weapon (7–9). It is one of the most infectious pathogenic bacteria known; it can be aerosolized; few antibiotics are approved for treatment; and antibiotic-resistant strains can be readily constructed or spontaneously generated in the process of antimicrobial sensitivity testing (10–15). In addition, there is no licensed vaccine against tularemia currently available. Thus, the use of *F. tularensis* in an aerosol biological attack remains a threat to society (8, 16). Such events or other modes of intentional release would likely result in environmental contamination with *F. tularensis*, increasing the need to understand mechanisms by which it can persist in the environment (17).

As tularemia does not transmit person to person, all human cases are independently acquired from environmental sources. Therefore, understanding *F. tularensis* persistence in the environment is important for predicting and controlling natural human disease events. Human cases caused by the highly virulent *F. tularensis* subsp. *tularensis* (hereafter, type A), which occurs only in North America, have been primarily associated with exposure to lagomorphs, ticks, and biting flies in comparatively dry environments. In contrast, cases caused by the less virulent *F. tularensis* subsp. *holarctica* (hereafter, type B), which occurs throughout the northern hemisphere, have been primarily associated with exposure to ticks, mosquitoes, streams, ponds, lakes, rivers, as well as exposure to lagomorphs and rodents, including aquatic rodents such as muskrats and beavers (18, 19). However, beyond these broad associations, the natural cycle and perpetuation of *F. tularensis* in the environment have proven extremely difficult to delineate, as it appears to persist in distinctive regional ecologies involving different transmission routes to multiple mammal species that can become infected with *F. tularensis*, as well as a variety of potential arthropod vectors (9, 20–25). The absence of cohesive evidence for defined refugia or specific disease reservoirs, as well as a lack of knowledge about specific mechanisms that facilitate long-term persistence in the environment between infection events in mammals, continues to make disease prevention challenging (25, 26).

The capability of *F. tularensis* to cause disease in many different small to medium mammal species likely accounts for an important role in the perpetuation of *F. tularensis*—as a means to produce and then seed large numbers of cells into the environment (27, 28). Laboratory infection experiments in mice have shown a bacterial load ranging between 10^9^ and 10^11^ bacteria per mL of blood or per gram of tissue (29, 30). Indeed, carcasses of animals such as voles and beavers, deceased from tularemia and containing high bacterial burdens, are known to contaminate local aquatic environments (28, 31–35). Multiple studies suggest that persistence in an aquatic environment is possible for *F. tularensis*; this is not surprising given its closest known relative, *F. novicida*, is almost exclusively associated with water (36). Berrada and Telford (37) found that brackish water prolonged the persistence of both types A and B *F. tularensis*, and multiple studies have proposed additional mechanisms for type B persistence in aquatic environments. These include as planktonic (free living in water) bacteria, or on or within aquatic hosts, such as protozoa, arthropods (mosquito larvae), mollusks, or crustaceans (30, 38–46). In addition, type B has been shown to successfully establish micro-colonies within preformed, aquatic multi-species *ex-vivo* biofilm in cold water (4°C) (46). However, neither type A nor type B wild-type strains form intrinsic (i.e., single species) biofilms in water at either 4°C or 20°C (47).

In this work, we investigated the time window for planktonic persistence as a potential mechanism for *F. tularensis* survival between infection events in mammals. In our study design, we defined persistent *F. tularensis* as cells that remained capable of causing tularemia in mice, and we examined how this persistence varied across different aquatic conditions, including variations in temperature, nutrient availability, salinity, and bacterial density. Importantly, these experiments were conducted using fully virulent strains of both type A and type B to facilitate direct comparisons between these two disease-causing subspecies.

## RESULTS

**Fig 1:**
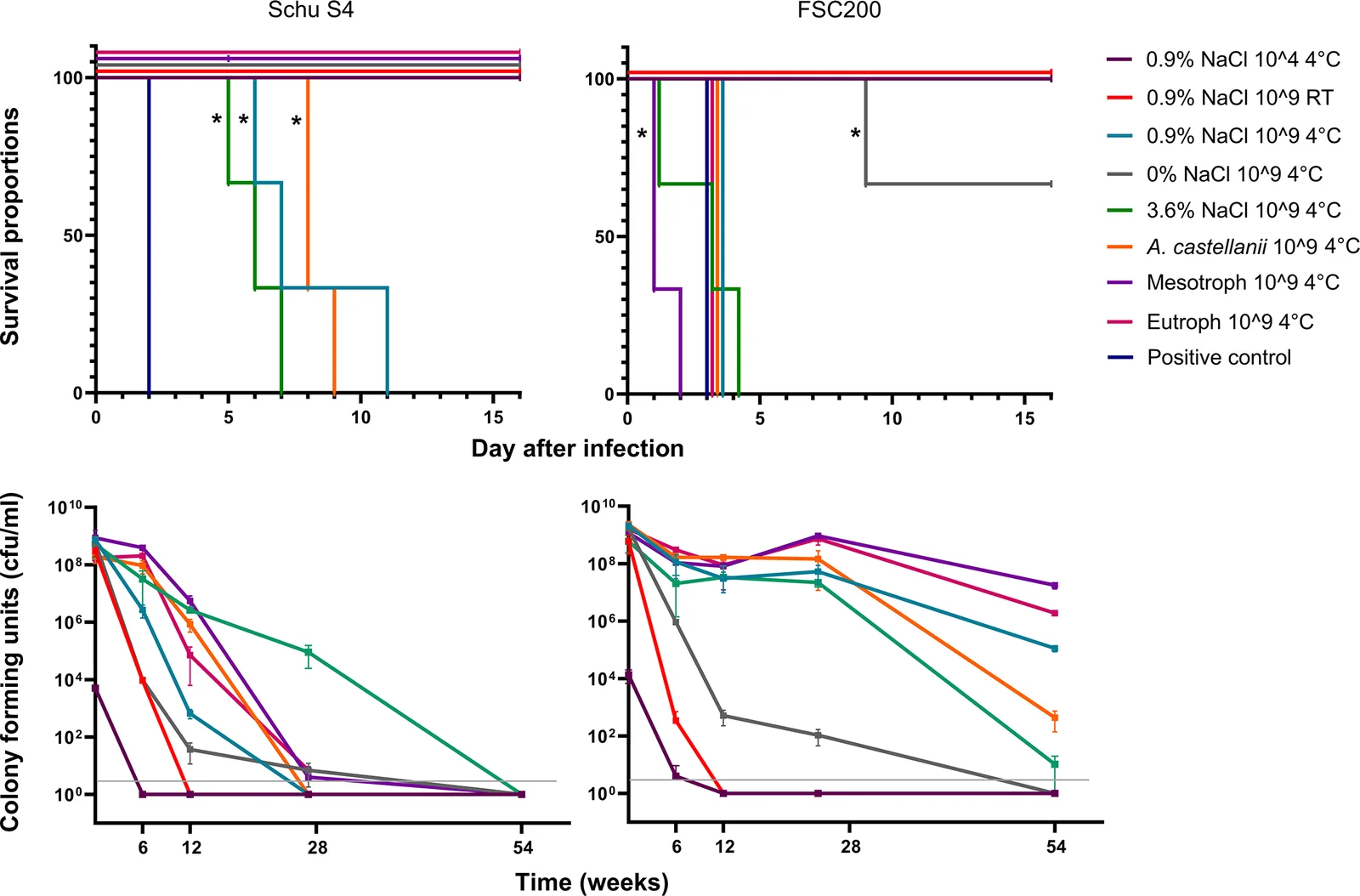
Upper panels: Proportion of surviving mice (*y*-axis, *n* = 3/treatment) across time (*x*-axis) infected by inoculum of *F. tularensis* Schu S4 (left) and FSC200 (right) obtained after 54 weeks from eight different aquatic treatments (Table 1) and actively growing controls (10^2^ CFU of Schu S4 and FSC200, respectively). Each non-negative event represents a mouse confirmed with tularemia using culture and PCR. The treatments (listed in the legend and Table 1) included two inocula (10^4^ and 10^9^ CFU/mL), two temperatures (RT and 4°C), two changes to NaCl concentration (from 0.9% to 0% or 3.6%), the addition of amoeba (10^4^ A. castellanii), and two additional levels of nutrient availability (C:N:P addition to mesotrophic and eutrophic conditions). Survival curves indicated with asterisks yielded statistically significant (*P* < 0.05) differences in onset of disease compared to positive controls. Lower panels: Viable counts (*y*-axis) of planktonic Schu S4 (left) and FSC200 (right) strains in water collected from the eight treatments across five time points spanning >1 year (54 weeks; *x*-axis). Viable counts are presented as colony-forming units (CFUs/mL). Bars and whiskers in each graph represent data from sampling three biological replicates from each treatment at each time point. Gray lines in graphs represent limits of detection for the analyses.

To investigate the ability of planktonic *F. tularensis* to survive long-term*,* we conducted a 54-week study evaluating persistence of virulent *F. tularensis* strains Schu S4 (type A) and FSC200 (type B) in axenic aqueous conditions. We utilized and compared eight treatments (Table 1) to identify specific environmental conditions that may have an effect on persistence, including differences in density of the starting inoculum (10^4^ and 10^9^), temperature (room temperature [RT] and 4°C), difference in salinity (0%, 0.9%, and 3.6% NaCl), three levels of nutrients (corresponding to oligotrophic [low; no added nutrients], mesotrophic [medium], and eutrophic conditions [high]), and presence/absence of amoeba (*Acanthamoeba castellanii*) (Table 1). We monitored persistence via the ability to cause disease in a mouse model (Fig. 1A) but also observed viable counts on GC II solid agar (as CFUs) (Fig. 1B). In all cases, we confirmed that disease symptoms were caused by *F. tularensis* via positive culture from mouse spleens and subsequent *F. tularensis*-specific PCR. We estimated *F. tularensis* cell integrity by measuring total cell counts (via flow cytometry, confirmed with qPCR) together with cell membrane integrity via the addition of propidium monoazide (PMA) followed by an additional qPCR assay (PMA-PCR) (Table 1; Fig. S1). PMA binds to DNA and prevents freely available DNA to be amplified in the additional PCR. Thus, the signal reduction effect after PMA treatment was expressed as “damaged bacteria” obtained by subtraction of quantification cycle threshold (Ct) values of non-PMA-treated cell suspensions from the Ct values of the corresponding PMA-treated cell suspensions (Table 1).

**TABLE 1:** Summary table of axenic long-term (54 weeks) treatments of *F. tularensis* Schu S4 and FSC200 strains^*b*^

Strain	Temperature (°C)	Starting density cells/mL (log)	NaCl addition (%)	Nutrient addition	*A. castellanii* presence	Total counts at 54 weeks cells/mL (log)	Damaged bacteria at 54 weeks (log)^*a*^	Culturable at 54 weeks	Virulence in mice at 54 weeks
Schu S4	4	**1 × 10^4^ (4.0**)	0.9%	−	Absent	bd	bd	−	−
	**RT**	1 × 10^9^ (9.0)	0.9%	−	Absent	3.1 × 10^8^ (8.5)	1.4	−	−
	4	1 × 10^9^ (9.0)	0.9%	−	Absent	5.9 × 10^8^ (8.8)	<1	−	**+**
	4	1 × 10^9^ (9.0)	**0%**	−	Absent	6.4 × 10^8^ (8.8)	<1	−	−
	4	1 × 10^9^ (9.0)	**3.6%**	−	Absent	3.6 × 10^8^ (8.6)	1.7	−	**+**
	4	1 × 10^9^ (9.0)	0.9%	−	**Present**	1.0 × 10^9^ (9.0)	1.4	−	**+**
	4	1 × 10^9^ (9.0)	0.9%	**Mesotrophic**	Absent	1.2 × 10^9^ (9.1)	1.2	−	−
	4	1 × 10^9^ (9.0)	0.9%	**Eutrophic**	Absent	1.3 × 10^9^ (9.1)	2.1	−	−
FSC200	4	**1 × 10^4^ (4.0**)	0.9%	−	Absent	bd	bd	−	−
	**RT**	1 × 10^9^ (9.0)	0.9%	−	Absent	1.3 × 10^8^ (8.1)	<1	−	−
	4	1 × 10^9^ (9.0)	0.9%	−	Absent	8.4 × 10^7^ (7.9)	<1	**+**	**++**
	4	1 × 10^9^ (9.0)	**0%**	−	Absent	2.1 × 10^8^ (8.3)	<1	−	**+**
	4	1 × 10^9^ (9.0)	**3.6%**	−	Absent	1.3 × 10^8^ (8.1)	<1	**+**	**++**
	4	1 × 10^9^ (9.0)	0.9%	−	**Present**	9.3 × 10^7^ (8.0)	<1	**+**	**++**
	4	1 × 10^9^ (9.0)	0.9%	**Mesotrophic**	Absent	1.3 × 10^8^ (8.1)	<1	**+**	**++**
	4	1 × 10^9^ (9.0)	0.9%	**Eutrophic**	Absent	1.4 × 10^8^ (8.1)	<1	**+**	**++**

^*a*^ ΔC_t_ qPCR = 3 corresponds to a 1-log difference in the number of bacteria.

^*b*^ Treatment conditions (*n* = 8) include variations in (i) starting density of bacteria, (ii) temperature, (iii–v) NaCl level, (vi) presence of amoeba (*Amoeba castellanii *10^4^ amoeba), and (vii and viii) addition of nutrients (carbon, nitrogen, and phosphorus [CNP] addition corresponding to mesotrophic and eutrophic conditions); values distinct from other treatments are indicated with bold text. The *F. tularensis* populations were characterized at 54 weeks for total cell count (cells/mL; bd, below detection limit of the assay), estimate of cell damage using PMA treatment before qPCR (log damaged bacterial cells as inferred from the ΔC_t_ value obtained), culturability on solid GC II agar (+, bacterial growth; −, no growth), virulence in mice (++, all mice within a treatment died within 5 days; +, at least one mouse within the treatment died and onset of disease occurred ≥5 days after infection; −, no mice displayed symptoms of disease within 16 days).

### Both Schu S4 and FSC200 can persist for >1 year outside a host

Both subspecies were able to persist for 54 weeks under multiple aquatic treatment conditions. After 54 weeks, Schu S4 inoculum from three treatments and FSC200 inoculum from those same three treatments plus three others (six total) still led to tularemia in some or all challenged mice (Fig. 1A). In addition, FSC200 populations from five treatments were still culturable after 54 weeks; however, none of the Schu S4 populations were culturable at 54 weeks (Fig. 1B). The smaller number of treatments that remained culturable compared to those still capable of causing disease in mice after 54 weeks, which was the case across both subspecies, indicates that some of these populations had entered a viable but nonculturable (VBNC) state and confirms that the mouse model is essential for identifying persistent populations. In general, CFUs decreased as a function of time across all treatment conditions and both subspecies.

In the three treatments in which both Schu S4 and FSC200 retained virulence after 54 weeks (0.9% NaCl, 3.6% NaCl, and *A. castellanii* present), time to disease onset was far shorter for FSC200 compared to Schu S4, and this difference was correlated with culturability, as FSC200 was cultured from all three of these treatments at 54 weeks, but Schu S4 was not. Onset of disease was observed 1–4 days (Fig. 1A) after mice were inoculated with all five of the 54-week FSC200 treatments that were still culturable (Fig. 1B); this is comparable to infection doses of 10^1^ or 10^3^ of actively growing populations of the same strain, which caused disease onset before day 4 (Fig. S2). In contrast, the three 54-week non-culturable Schu S4 treatments that retained virulence resulted in a statistically significant delay in the onset of disease in mice (Fig. 1A) compared with actively growing cells of this same strain (*P* < 0.05 for all pairwise combinations). Of note, this delay in disease onset after infection (>5 days) cannot be explained as an effect of low inoculum, as the lowest possible infectious doses of actively growing Schu S4 (10^0^ and 10^1^) both still resulted in disease onset at day 3 post-infection (Fig. S2).

Persistence of both Schu S4 and FSC200 after 54 weeks, as determined by causing disease in mice, was enhanced by a higher starting inoculum, cold temperatures, and at least some level of salinity, but the presence of *A. castellanii* seemed to have no effect on persistence of either strain past 54 weeks (Fig. 1; Table 1). Lower density starting populations (10^4^ cells/mL) of both subspecies incubated at 4°C yielded viable count, total count, and qPCR results that were below the limits of detection already at week 6 (data not shown) and were unable to cause tularemia in mice at 54 weeks (Table 1). Similarly, with NaCl held constant at 0.9%, the treatment maintained at RT also resulted in a quick reduction of CFUs and no disease in mice at 54 weeks for either Schu S4 or FSC200 compared to the same treatment at 4°C; the latter resulted in full virulence in mice at 54 weeks for both subspecies. The lower temperature also prolonged culturability of both strain types: viable counts for Schu S4 incubated in 0.9% NaCl solution showed growth (i.e., CFUs > 0) for at least 12 weeks at 4°C compared to 6 weeks at RT, and FSC200 incubated in 0.9% NaCl solution at 4°C still displayed growth (10^5^ CFU/mL) at 54 weeks. With temperature held constant at 4°C, persistence of both Schu S4 and FSC200 at 54 weeks decreased dramatically in the treatment with 0% NaCl (i.e., ultrapure water with no addition of NaCl) compared to the treatments with 0.9% or 3.6% NaCl, with a complete loss of viable counts for both subspecies and disease in only one of three mice challenged with the 54-week 0% NaCl FSC200 inoculum and none of the mice challenged with Schu S4. The increased salinity in the 3.6% NaCl treatment compared to the 0.9% NaCl treatment slightly reduced FSC200 viability counts at 54 weeks, but inocula from both treatments at 54 weeks still rapidly caused disease in mice. In contrast, culturability of Schu S4 was extended to at least 28 weeks in the 3.6% NaCl treatment compared to at least 12 weeks in the 0.9% NaCl treatment. When NaCl and temperature were held constant at 0.9% and 4°C, respectively, the presence of *A. castellanii* had no perceptible effect on persistence of either Schu S4 or FSC200, as 54-week populations from both of these treatments and from both subspecies remained virulent in mice; viable counts were also similar for both subspecies in the presence or absence of *A. castellanii*.

Schu S4 and FSC200 responded differently to the addition of nutrients (in the form of increased amounts of added organic C and inorganic N and P) when salinity and temperature were held constant at 0.9% NaCl and 4°C, respectively. Schu S4 viable counts dropped to zero after 12 weeks in all three treatments (Fig. 1), with greater cell damage in the treatments with increased nutrients (Fig. S1). In addition, mice challenged with 54-week inoculum from the oligotrophic treatment (i.e., 0.9% NaCl, 4°C, and no added nutrients) all succumbed to disease, whereas inocula from the 54-week Schu S4 mesotrophic and eutrophic treatments did not result in disease in any of the challenged mice. In contrast, the addition of nutrients had little impact on persistence of FSC200, with viable counts remaining consistent across the three treatments and only a slight decrease in CFUs at 54 weeks; cell damage was also minimal across all three treatments (Fig. S1). Inoculum from all three FSC200 treatments also remained virulent in mice.

Measurements of total cell counts via flow cytometry (and confirmed by qPCR) indicated the presence of intact cells after 54 weeks for both Schu S4 and FSC200 across all treatments, with levels similar to the starting inocula, but PMA-PCR revealed significant signs of cell damage starting at 12 weeks in all Schu S4 treatments except the 0% saline at 4°C treatment; cell damage was not observed for any of the FSC200 treatments (Fig. S1). Losses in total cell counts were observed for FSC200 in several treatments, but this was a reduction of only one log, from an initial bacterial starting population of 10^8^–10^9^ cells/mL to 10^7^–10^8^ cells/mL after 54 weeks; Schu S4 total cell counts stayed essentially the same across the 54 weeks. The PMA-PCR results suggested cell damage across all Schu S4 treatments, with the largest proportion of available free DNA (indicating cell damage) at the 12-week sampling point. At that point, free DNA in the Schu S4 treatments was measured as ΔCt corresponding to a 3.3 log reduction of bacteria in the 3.6% NaCl incubation, a 2.6 log reduction of bacteria in mesotrophic conditions, a 2.3 log reduction of bacteria in eutrophic conditions, and a 3.1 log reduction of bacteria in the presence of *A. castellanii*. In contrast, PMA-PCR results for FSC200 yielded ΔCt values corresponding to less than a 1 log reduction of bacteria across all treatments and at all time points, indicating mostly intact cells over the 54 weeks.

### FSC200 can remain virulent after 85 weeks in cold water

Because both strains persisted to 54 weeks in multiple treatments, we conducted a second, longer experiment to: (i) determine if the previous results were reproducible, (ii) further delineate the temporal limits of persistence, and (iii) determine the proportion of bacteria persisting within populations. This experiment was similar to the 54-week study with the exceptions that incubation was prolonged to 85 weeks, only one of the treatment conditions from the previous experiment was employed (0.9% NaCl incubated at 4°C), and strains Schu S4 *gfp lux* and FSC200 *gfp lux* were utilized (see Materials and Methods). These strains were constructed to facilitate activity measurements and for use in more complex pond water incubations (see next section) but were otherwise indistinguishable from the wild-type parental strains with regard to growth in culture medium, macrophage infection, virulence in mice, and persistence during co-culture with *A. castellanii* in water (Fig. S2 and S3).

**Fig 2:**
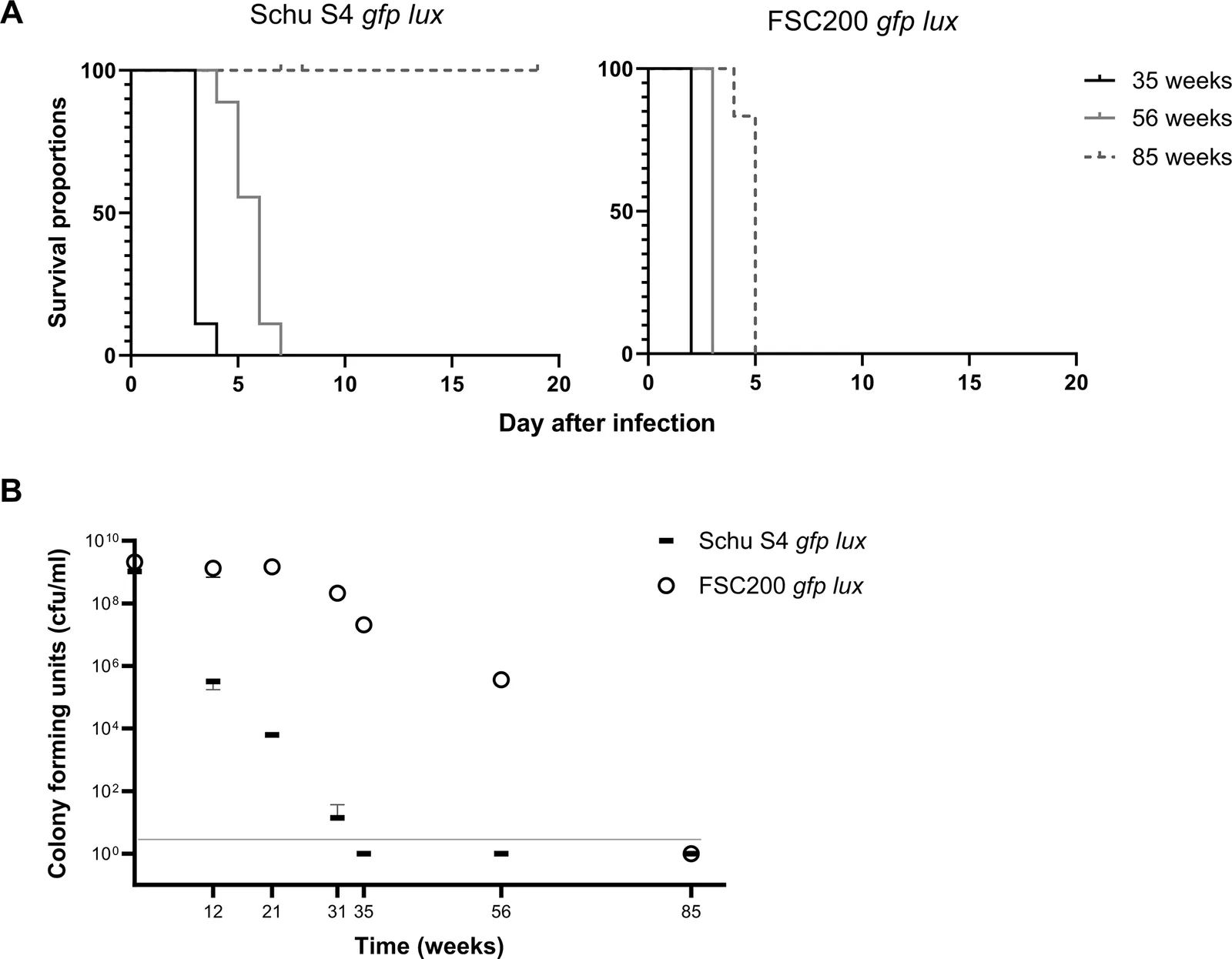
(**A**) Survival of mice (*n* = 4/treatment) infected by Schu S4 *gfp lux* (left) and FSC200 *gfp lux* (right) populations after 35, 56, and 85 weeks. Each non-negative event represents a mouse confirmed with tularemia. Of note, FSC200 *gfp lux* at 35 and 56 weeks display survival curves for mice infected with populations that were still culturable on solid GC II agar. The other four survival curves are for mice infected with bacterial populations with no growth on this agar. Survival curves are significantly different in pairwise comparison to the other timepoints in the timeseries (*P* < 0.05) via Log rank test. (**B**) Viable counts (presented as CFUs per milliliter on solid GC II agar) of strains Schu S4 *gfp lux* and FSC200 *gfp lux* in 0.9% NaCl and 4°C at sampling time points 0, 12, 21, 31, 35, 56, and 85 weeks. Symbols and error bars represent the mean and standard deviation using three biological replicates. The gray line in the graph represents the limit of detection for the culture-based analysis. A significantly slower decline of CFUs for FSC200 *gpf lux* compared to Schu S4 *gfp lux* (*P* < 0.05) was confirmed by an unpaired *t*-test of area under curve values for the biological replicates.

We were able to replicate the 54-week study and determine the temporal limit of Schu S4 *gfp lux* to perpetuate disease under these conditions; it persisted for at least 56 weeks but less than 85 weeks (Fig. 2A). As in the 54-week experiment (Fig. 1), CFUs of Schu S4 dropped significantly more rapidly than for FSC200 (*P* < 0.05, Fig. 2B). Despite the lack of culturability of Schu S4 *gfp lux* at 35 weeks, inoculum from the Schu S4 *gfp lux* incubation at the 35- and 56-week time points were still capable of causing disease in all of the challenged mice, albeit with significant delays in disease onset (*P* < 0.05 for all pairwise combinations) compared to actively growing populations of the same strain (Fig. 2A; Fig. S2).

In contrast, FSC200 *gfp lux* remained virulent in mice even at 85 weeks (Fig. 2A), leading to disease in all challenged mice, which demonstrates that the outer temporal limits for it to persist under these conditions have not yet been determined. Viable counts for FSC200 *gfp lux* dropped throughout the experiment and were last observed at the 56-week sampling point, with no CFUs at 85 weeks (Fig. 2B). Disease onset in mice was again correlated with culturability, as inocula from the FSC200 *gfp lux* 35-week and 56-week populations, when there were still culturable bacteria in the populations, led to rapid disease onset in mice similar to that of actively growing populations of the same strain (Fig. 2A). In contrast, disease onset was significantly delayed in the 85-week FSC200 *gfp lux* populations that were no longer culturable (*P* < 0.05 for all pairwise combinations). Taken together, these laboratory findings indicate that the main difference between Schu S4 and FSC200 persistence under these conditions is the rate of decline to extinction.

**Fig 3:**
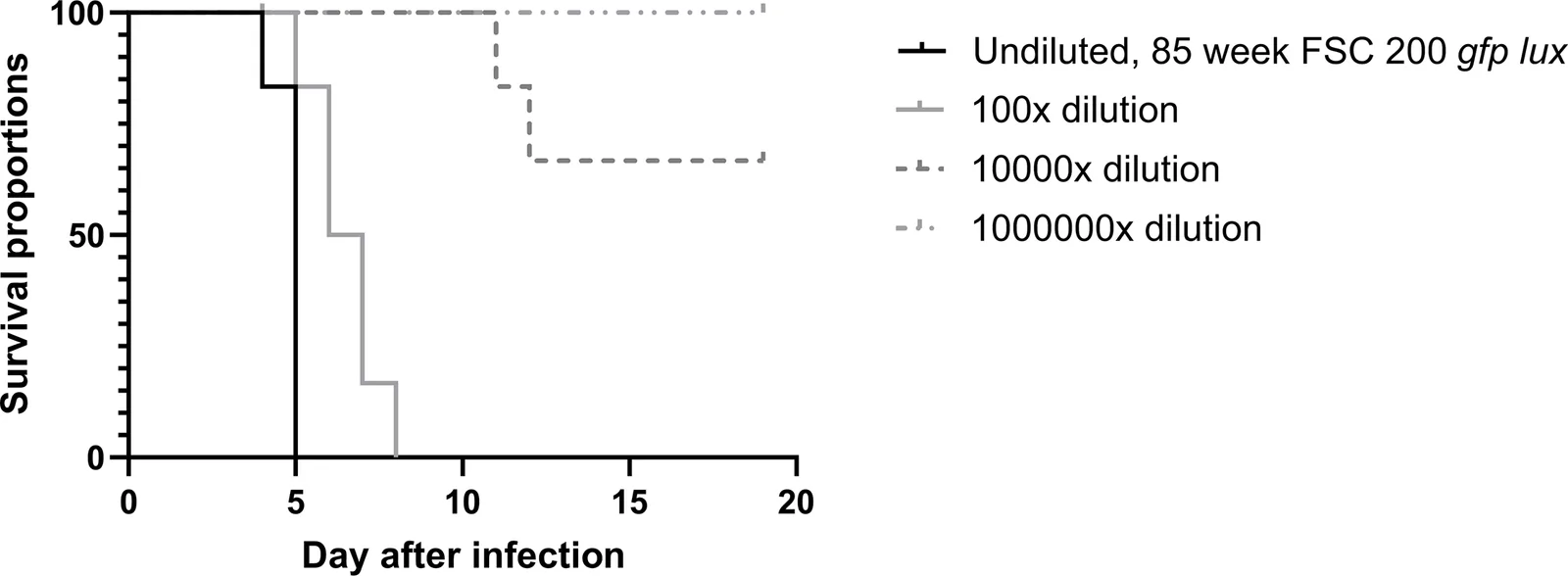
Survival of mice infected with a serial dilution of the FSC200 *gfp lux* populations after 85 weeks of incubation in saline solution (0.9% NaCl) at 4°C. Viable count analysis of the undiluted sample resulted in 0 CFU (Fig. 2A). Total count analysis of the undiluted sample was 8.8 × 10^8^ counts/mL (flow cytometry). Dilutions of the bacterial population incubated for 85 weeks were performed in 0.9% NaCl saline solution in three steps of 100× dilution in each step. Each non-negative event represents a mouse confirmed with tularemia.

Although many of the bacteria remained intact in the 85-week FSC200 *gfp lux* population, only a small proportion of them were still capable of causing disease in mice (Fig. 3). Flow cytometry analysis of undiluted sample from the 85-week FSC200 *gfp lux* population indicated there were still 8.8 × 10^8^ cells/mL, similar to the number of cells used to initiate the experiment. To determine the proportion of these non-culturable cells that were still capable of causing disease in mice, we performed a serial dilution of a sample from the 85-week population in 0.9% NaCl solution in three steps of 100× dilution (i.e., 10^2^×, 10^4^×, and 10^6^×) and used these dilutions as inocula for mouse challenges. Compared to undiluted inoculum from the 85-week population, these diluted inocula resulted in delayed disease onset (10^2^× dilution), disease in only a subset of the challenged mice (10^4^× dilution), or no disease in mice (10^6^× dilution; Fig. 3). As the 10^4^× dilution was the most diluted sample still capable of causing disease in mice, and assuming a single viable cell of *F. tularensis* can cause disease in a mouse (Fig. S2), this suggests that only ~10^4^ cells in the 85-week population were still capable of causing disease in mice, corresponding to <0.01% of the actively growing cells that were used to initiate this experiment. Thus, only a small fraction of the original inoculum of FSC200 *gfp lux* persisted at 85 weeks. However, that small fraction constituted enough live bacteria to perpetuate disease.

### *F. tularensis* genome equivalents degrade more rapidly in natural pond water held at RT and containing a higher diversity of other microbes

To characterize *F. tularensis* persistence in aquatic conditions that mimic natural pond or lake water environments, including the presence of other bacteria and potentially predatory protozoa, we conducted another set of 10-week aqueous microcosm experiments using natural pond water (Table 2). Prior to the start of this experiment, we filtered the water into two fractions: 0.2 μm, which excluded all microbes in the picoplankton size and above, and 200 μm, which included pico-, nano-, and microplankton but excluded macroplankton. To mimic natural seeding density expected from decaying *F. tularensis*-infected animal carcasses in water, we inoculated our 100 mL microcosm samples with high inocula of the Schu S4 *gfp lux* (1.2 × 10^8^ CFUs/mL) and FSC200 *gfp lux* (2.6 × 10^8^ CFUs/mL) strains; three biological replicates were established for each strain at both RT and 4°C, and in both filtered fractions. To minimize the “bottle effect” (48), every second day, 10% of the volume of each sample was replaced.

**TABLE 2:** Summary table of the results from the natural pond water incubations of *F. tularensis* strains Schu S4 *gfp lux* and FSC200 *gfp lux* with starting inocula of 1.2 × 10^8^ and 2.6 × 10^8^ bacteria/mL, respectively^*a*^

Strain	Pond water screening filter size (µm)	Incubation temperature (°C)	Luciferase activity in pond water (week last observed)	Luciferase activity in response to growth media at 37°C (week last observed)	*F. tularensis* genome equivalents/mL after 8 weeks (log)	*F. tularensis* relative abundance in microcosm bacterial community after 8 weeks	Virulence in mice after 10 weeks
Schu S4 *gfp lux*	200	RT	No activity	No activity	bd	1.4%	–
FSC200 *gfp lux*	200	RT	No activity	No activity	1.1 × 10^3^ (3.0)	0%	–
Schu S4 *gfp lux*	0.2	RT	No activity	No activity	3.1 × 10^6^ (6.5)	16.7%	–
FSC200 *gfp lux*	0.2	RT	No activity	Week 6	6.3 × 10^6^ (6.8)	8.0%	–
Schu S4 *gfp lux*	200	4	No activity	No activity	1.4 × 10^6^ (6.1)	2.2%	+
FSC200 *gfp lux*	200	4	No activity	Week 2	2.9 × 10^5^ (5.5)	3.0%	++
Schu S4 *gfp lux*	0.2	4	No activity	Week 2	1.2 × 10^8^ (8.1)	80.0%	++
FSC200 *gfp lux*	0.2	4	No activity	Week 8	1.6 × 10^8^ (8.2)	83.2%	++

^*a*^ Treatment conditions (*n* = 4 per strain) included differences in pond water pre-filtration treatment and incubation temperature. Results include *F. tularensis* luciferase activity in pond water without nutrient addition, luciferase activity in response to growth media (Chamberlain defined medium) addition at 37°C, genome equivalents of *F. tularensis* at 8 weeks as inferred from qPCR data, *F. tularensis* relative abundance in the microcosm bacterial community based upon 16S rRNA gene analysis, and *F. tularensis* virulence in mice after 10 weeks of pond water incubation. ++, all mice within a treatment died within 5 days; +, at least one mouse within the treatment died and onset of disease 5 days after the day of infection or later; –, no disease in mice; bd, below limit of detection of the assay.

**Fig 4:**
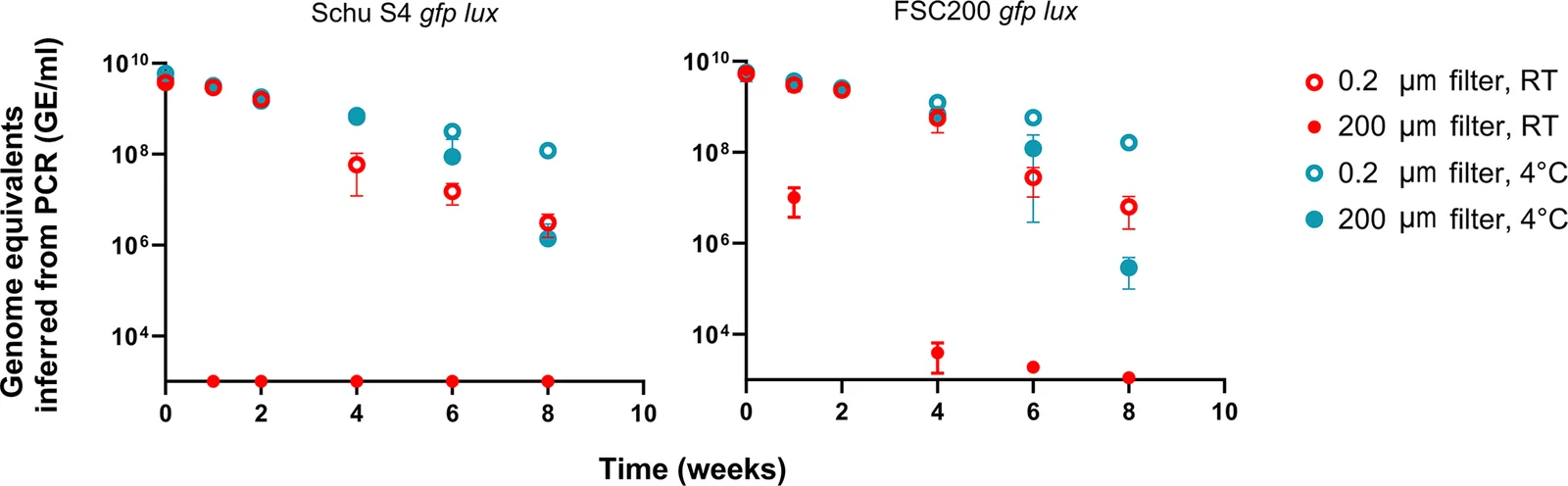
Mean bacterial abundance per milliliter pond water as genome equivalents inferred from an *F. tularensis*-specific TaqMan PCR assay (*y*-axis). *F. tularensis* genome equivalents in pond water passed through a 0.2 µm filter (open circles) or a 200 μm filter (filled circles), spiked with Schu S4 *gfp lux* (left panel) and FSC200 *gfp lux* (right panel), maintained at RT (red) or 4°C (blue), and measured at 0, 1, 2, 4, 6, and 8 weeks (*x*-axis). Note that Schu S4 *gfp lux* genome equivalents were below the limit of detection from week 2 in the 200 μm filtered pond water incubated at RT (left panel). The *F. tularensis* limit of detection for the PCR assay was <10^3^ bacteria/mL.

*F. tularensis* genome equivalents were estimated in the pond water incubations across the first 8 weeks using qPCR (Fig. 4). Across all data, genome equivalents declined significantly over time (*P* < 0.05 by analysis of covariance [ANCOVA]). Consistent with viable count analysis in the 54-week study, reductions in genome equivalents were significantly affected by temperature (time × temperature, *P* < 0.05), where a more rapid reduction in genome equivalents occurred within microcosms held at RT compared to 4°C. In addition, genome equivalents of both Schu S4 *gfp lux* and FSC200 *gfp lux* degraded more rapidly in the high microbe fraction (time × filter fraction, *P* < 0.05, Fig. 4). The rate of decline was not significantly affected by strain (time × strain *P* > 0.05). Thus, the reduction of *F. tularensis* genome equivalents was most pronounced in microcosms held at RT and containing more complex microbial diversity, where genomic copies of both strains fell below or close to the limit of detection (>7 log reduction) after 8 weeks (Fig. 4).

The microcosm samples were evaluated at 0, 1, 2, 4, 6, and 8 weeks using 16S rRNA amplicon sequencing to examine changes in the relative abundances of bacterial taxa at the family level, which revealed that the relative abundance of *F. tularensis* decreased in all treatments after 8 weeks. The 200 μm fractions of pond water held at RT shifted from a relative dominance of *F. tularensis* at time 0 to a complex microbial community within 1 or 3 weeks (Fig. S4). There was still an increase in the relative abundance of other bacteria in the 0.2 μm fractions and in water held at 4°C across the 8-week experiment, but with less diverse taxonomic composition (families) compared to the 200 μm fraction (Fig. S4).

### Both subspecies are metabolically inactive in natural pond water

*F. tularensis* metabolic activity was monitored at six time points (0, 1, 2, 4, 6, and 8 weeks) via luciferase measurements directly from the microcosms. Luciferase emits light but only in the presence of bacterial ATP and, thus, allows quantification of metabolically active cells (Fig. S5); it is encoded by the *lux* operon (*lux CDABE*) of strains Schu S4 *gfp lux* and FSC200 *gfp lux*. We did not measure any luciferase activity in any of the treatments in our 10-week experiment (Table 2), and this was consistent across the two subspecies, different temperatures (RT vs. 4°C), and the low versus high microbe fractions.

### Persisting *F. tularensis* cells that can still cause disease in mice are outcompeted by other bacteria in laboratory culture conditions

**Fig 5:**
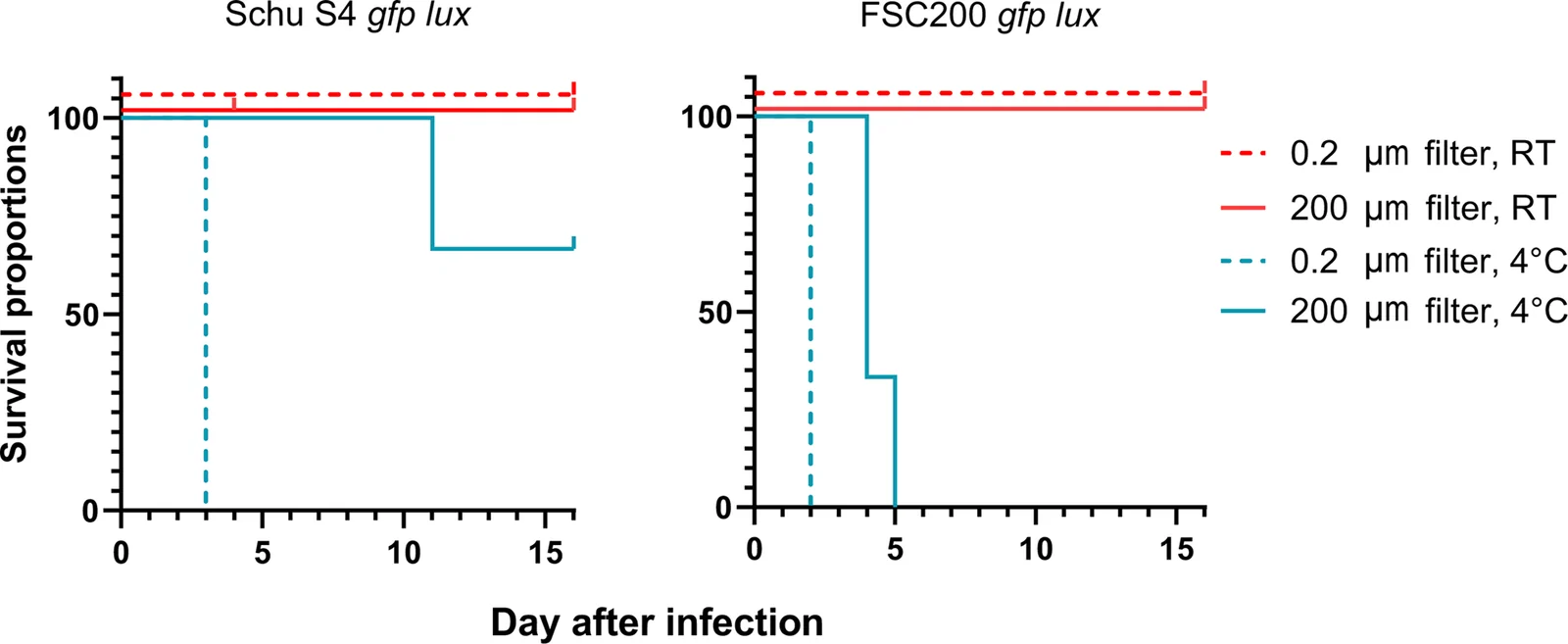
Survival proportions of mice infected with *F. tularensis* strains Schu S4 *gfp lux* (left) and FSC200 *gfp lux* (right) after 10 weeks in the pond water incubations (0.2 µm filter eluate dotted lines and 200 µm eluate solid lines). All mice survived inoculation with *F. tularensis* populations from pond-water incubated at RT (red), whereas the majority of mice did not survive inoculation with *F. tularensis* populations from pond-water incubated at 4°C (blue). The observed difference in survival curve for mice infected with Schu S4 *gfp lux* in 0.2 µm filtered eluate at 4°C was significantly different in pairwise comparison to the other treatments (*P* < 0.05). The survival curve for mice infected with FSC200 *gfp lux* in 200 µm filter eluate at 4°C was significantly different from 0.2 µm filter eluate at the same temperature and both RT incubations in pairwise comparisons.

We evaluated persistence by the ability of inocula from 10-week populations to cause tularemia in mice. All 10-week inocula containing either strain (Schu S4 *gfp lux* and FSC200 *gfp lux*) held at 4°C caused tularemia in mice, indicating that we still have not determined the outer temporal limits for either strain to persist under cold natural pond water conditions. In contrast, no inocula from microcosms held at RT caused disease (Fig. 5; Table 2). Disease onset was significantly delayed in the 4°C inocula containing other microbes compared to the 4°C inocula without other microbes (Fig. 5), likely because the *F. tularensis* population in these samples was closer to extinction (Fig. 4; Table 2).

Both Schu S4 *gfp lux* and FSC200 *gfp lux* were rapidly outcompeted by other microbes in growth media. We first examined axenic inoculations of both strains into liquid Chamberlain’s growth media, which revealed rapid growth within just a few hours, as measured by luciferase activity (Fig. S5). We then examined samples that were withdrawn from the microcosm experiments minutes after initiation (time 0) and then inoculated into Chamberlain’s media at 37°C (Fig. S6). Luciferase signal was observed starting at 8 h and increased until 14 h for both strains and from both water fractions. The luciferase signal for both strains in 0.2 µm pond water fraction continued to increase and remained measurable throughout the 38-h experiment, with growth curves similar to the axenic experiment (Fig. S5). But starting at 16 h, the luciferase signal for both strains in the 200 µm fraction started to decrease, dropping to 0 at 20 h, demonstrating that the increased microbial complexity in the 200 µm fraction had a negative effect on the ability of both subspecies to grow in rich liquid media at 37°C. We performed similar experiments with 1-, 2-, 4-, 6-, and 8-week inocula from the microcosm experiments, and only FSC200 *gfp lux* held at 4°C in the 0.2 µm fraction produced luciferase signal in rich liquid media at 37°C at the final 8-week timepoint (Table 2). As all four of the 10-week inocula still caused disease in mice (Fig. 5), this finding demonstrates that *F. tularensis* persisting in pond water is not outcompeted by other bacteria when introduced to mice, even when it represents a small proportion of the total bacterial population (Table 2) but is outcompeted in nutrient-rich media. The latter may be due to an inability of *F. tularensis* to effectively compete for readily available nutrients.

## DISCUSSION

The persistence of *F. tularensis* in natural water sources has been well documented (31, 49–51). In this study, we directly compared type A and type B strains and examined the results of viability assays, metabolic activity measurements, and the capability to cause tularemia in mice. We used both axenic conditions and complex natural water in our experiments. These well-defined long-term experiments with rigorous follow-up demonstrated that cold, oligotrophic natural waters contaminated with *F. tularensis* could remain a source for disease perpetuation for more than a year, thereby enabling overwinter persistence outside a host in geographic areas with cold seasons (47, 52).

Although we have identified cold, oligotrophic conditions as the main drivers of persistence in this study, it is possible that additional factors not examined here, such as association with refuges like protozoa or preformed bacterial biofilms (30, 40–43, 50), may also contribute to extended persistence under natural conditions. The longer that planktonic populations of *F. tularensis* can persist outside a mammalian host, the greater the likelihood that they can find new hosts and amplify via small mammal epizootics, and also serve as sources for human infection (28, 31, 53). Indeed, natural waters that experience periods of cooling are reported to be the source of water-borne human tularemia in areas such as Turkey, Norway, and Sweden (50). Turkey stands out for regularly reporting a high prevalence of water-borne tularemia (oropharyngeal form) (54), and large outbreaks of water-borne tularemia have occurred there (55–57). Notably, many of these outbreaks have occurred in the winter months and were associated with cold, oligotrophic spring water, further supporting the idea that lower temperature and low nutrient levels are significant factors in water-borne tularemia epidemiology.

The findings in this study contribute to our understanding of conditions favoring host-independent survival of *F. tularensis* in aquatic environments as a component of the perpetuation of tularemia. We previously established that persistence of both type A and type B outside a mammalian host under axenic, low-nutrient aquatic conditions was extended at lower temperatures (4°C), up to 6 months for type B and 3 months for type A (47). Here, we demonstrate that type B can persist for >1.5 years (at least 85 weeks) and type A for >1 year (at least 56 weeks) under the most favorable axenic aquatic conditions, which include cold temperature (4°C), salinity levels that commonly characterize natural water sources, and a higher starting density of cells. In pond water with naturally occurring microbial communities, *F. tularensis* retained virulence in mice for at least 10 weeks, with virulence retained in both subspecies at 4°C (but not RT) independent of the complexity of the microbial community. However, both strains degraded more rapidly in the presence of the more complex microbial community.

We determined that both subspecies persist in natural water in a metabolically inactive state, which may allow *F. tularensis* to persist in the presence of other microbes because it does not need to compete for nutrients within an active microbial food web and may be less susceptible as a target for predation by other microbes (i.e., protozoans) (58). This may be especially relevant in oligotrophic freshwater, where available nutrients are limited. As documented in this study and also by other researchers (59), *F. tularensis* is rapidly outcompeted by other bacterial species in culture. Multiple lines of genomic evidence support that *F. tularensis* remains metabolically inactive outside a mammalian host: ongoing genome erosion has led to the deletion of metabolic pathway genes (2, 3), a degenerated CRISPR/Cas system (60), and the absence of fully functional phage sequence insertions within its bacterial genome (only prophage remnants); also, there is little evidence of recombination (61). These genomic features suggest a life cycle with restricted competition and little association or contact with other microorganisms. Nevertheless, as demonstrated here, metabolically inactive *F. tularensis* in natural pond water can become active again upon introduction into a mammalian host, highlighting that this species is evolutionarily specialized for growth in mammals.

**Fig 6:**
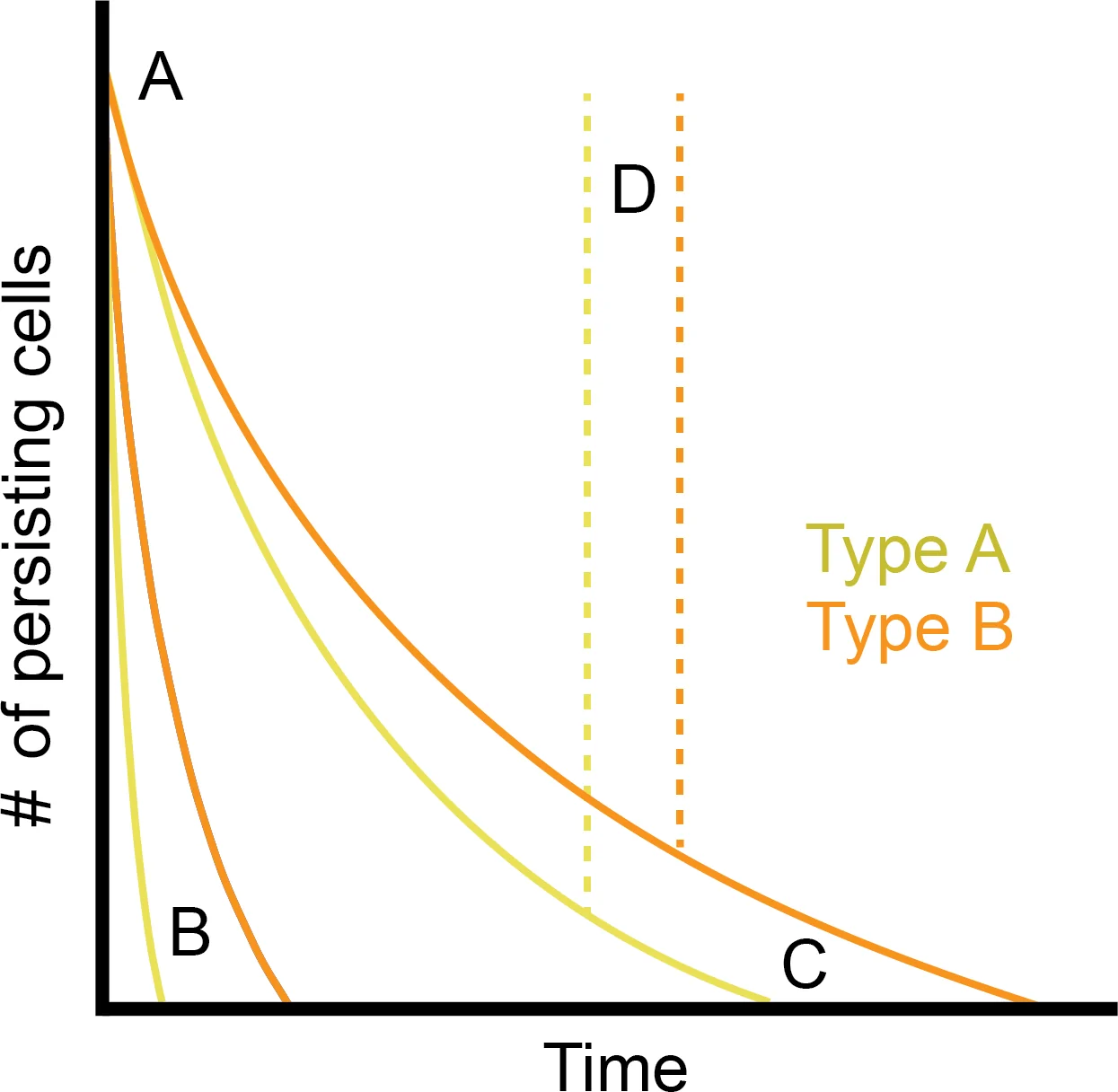
Decay path to extinction. The death of a mammal from tularemia deposits a large number of *F. tularensis* cells into an aquatic environment (**A**). Because *F. tularensis* is not replicating in these conditions, the number of persisting cells can only decrease through time. This decay will be more rapid in less favorable conditions (**B**), such as higher temperature and presence of other microbes, and will be more arrested in favorable conditions (**C**); the rate of decay is always more rapid for type A compared to type B, regardless of conditions. But even in the most favorable conditions, all cells in the starting population will eventually go extinct unless an infection is established in a new mammalian host, thereby restarting the cycle (**D**).

Lack of evidence for metabolic activity suggests that *F. tularensis* is not replicating in natural aquatic environments between disease events in mammals. This is supported by genomic epidemiological investigations that find very low or undetectable single nucleotide polymorphism (SNP) accumulation rates from repeated sampling of local *F. tularensis* phylogenetic lineages over time (53, 62–64). Thus, once a concentrated population of *F. tularensis* is deposited into an aquatic environment via the death of a mammalian host, its population size can only decrease and not increase, and it is, therefore, on a decay path to extinction (Fig. 6). In the beginning, there are still culturable bacteria present in the population that retain the ability to rapidly cause disease in mammals. This is followed by a loss of culturability but still the capability to cause disease in mice (i.e., a VBNC state), albeit with a delayed onset of disease; a VBNC state has been described in many diverse non-spore-forming bacterial species, including *F. tularensis* (41) and other pathogens (58, 65–67). However, the VBNC condition is a transient persistence state for *F. tularensis* along the decay path because, finally, even the capability to cause disease in mammals is lost as all cells in the starting population go extinct. Prior to total extinction, a particular lineage of *F. tularensis* can be rescued at any point along the decay path via infection in a new mammal host. The likelihood of infecting a new mammalian host is increased by the abilities of *F. tularensis* to (i) cause disease from a small inoculum (as few as 1–10 cells via the aerosol route), (ii) infect a wide diversity of rodent hosts (20), and (iii) be transmitted to mammalian hosts via multiple exposure routes (i.e., ingestion of contaminated water, inhalation, and arthropod bite). Of note, based on our findings, the decay path in water is steeper for type A compared to type B.

Not all aqueous conditions tested here maintained virulent *F. tularensis*. Some non-culturable populations failed to cause disease in mice (Fig. 1; Table 1) despite evidence of viability based on *in vitro* assay measurements (i.e., PMA PCR assay, Fig. S1). Thus, *in vitro* measurements that infer viability do not always equate to retention of virulence (68). In addition, our study reveals how small differences in aqueous composition can strongly influence the duration of *F. tularensis* persistence. This may partially explain the conflicting reports from previous *F. tularensis* VBNC studies that used different test solutions: one study effectively demonstrated the capability of non-culturable type B to induce disease in mice (52), whereas subsequent studies failed to reproduce this outcome (38, 47, 69, 70). As shown here and discussed previously by others (23), differences in the specific strains utilized, study setup, and means of detection and methods used for characterization of *F. tularensis* populations can profoundly influence results.

Type B persisted longer than type A in all of our aquatic experiments and under a wider range of conditions. The transient signs of cellular damage (Fig. S1C), the changed response to nutrient addition (i.e., inability to grow on GC II agar plates) (Fig. 1B), and the delay in onset of disease in mice (Fig. 1A) observed in Schu S4 at week 54 but not until week 85 in FSC200 indicates that the two subspecies have different biological responses to identical conditions. The two strains had opposite responses to increased aquatic nutrient levels (mesotrophic and eutrophic), with persistence decreased in Schu S4 but enhanced in FSC200 (Fig. 1). Interestingly, relatively high salinity (3.6%) favored persistence of both FSC200 and Schu S4. Similar results have been reported from a previous microcosm study that suggested that the survival of environmental *F. tularensis* at Martha’s Vineyard, Massachusetts, may have been prolonged by tularemia carcasses sprayed with oceanic salt (37). Salt tolerance was also recently suggested by the detection of type B in several species of marine mammals (71, 72). On the lower end of the salinity scale, FSC200, but not Schu S4, persisted over a year in ultrapure water and 0% NaCl. Such tolerance to low osmolarity is likely conferred by a mechanosensitive channel recently described in type B strains; a channel demonstrated to protect *F. tularensis* from hypoosmotic shock and required for surviving the osmotic transition from a mammalian host to environmental waters (73). It is not known if the presence of this channel is responsible for the stronger association of type B strains with water (50). However, our study does strongly demonstrate a physiological difference between the two subspecies, with FSC200 being capable of long-term survival under broader aquatic conditions compared to Schu S4. These physiological differences could partially explain the different ecological niches and geographic distributions described for type A and type B.

In conclusion, data from this study on timespans of planktonic persistence of virulent *F. tularensis* yield new perspectives on the role and importance of an aquatic niche for this bacterium. The results highlight the differences between type A and type B strains. We suggest a perpetuation model where survival of planktonic virulent *F. tularensis* in the environment is possible in a non-replicative state during a prolonged time window. The survival time is dependent on the local environment, and thereafter, *F. tularensis* either is reintroduced to a mammal or goes extinct in the absence of an association or introduction to a susceptible host. Under such a model, reintroduction within a certain time window becomes vital for perpetuation of tularemia, and local environmental conditions are key for establishment of local foci of disease.

## MATERIALS AND METHODS

### Bacterial strains and growth conditions

All *Francisella tularensis* and *Escherichia coli* strains used in this study are listed in Table 3. *F. tularensis* strains were cultured on solid GC II Agar (Chocolate agar) with 1% Hemoglobin and 1% IsoVitaleX (9) or in liquid Chamberlain defined medium (74). The cultures were incubated at 37°C in 5% CO_2_ if not stated otherwise. *E. coli* strains were grown on blood agar base (Merck) plates or in Luria-Bertani broth at 37°C. Where appropriate for cloning plasmids, antibiotics were used at the following concentrations: kanamycin 50 µg/mL (*E. coli*) and 10 µg/mL (*F. tularensis*), tetracycline 15 µg/mL (*E. coli*) and 5 µg/mL (*F. tularensis*), and chloramphenicol 25 µg/mL (*E. coli*) and 2.5 µg/mL (*F. tularensis*). Preparation of plasmid DNA, restriction enzyme digests, ligations, and transformations into *E. coli* were performed as described previously (75). Laboratory work, including studies with virulent strains, was performed within a biosafety cabinet in biosafety level 3 laboratory. Quantification of culturable *Francisella* (viable count) was done by 10-fold dilutions of bacterial suspensions, and 100 µL from each sample was spread on GC II solid agar and incubated at 37°C in 5% CO_2_ as described above, followed by counting CFU per milliliter.

**TABLE 3:** Strains and plasmids used in the study

Strain or plasmid	Subspecies/description	Source/reference
*F. tularensis* strains		
Schu S4	*tularensis* (type A, subgroup A1), also known as FSC237	Human ulcer, 1941, USA (2, 76)
Schu S4 *gfp lux*	Schu S4 with (*gfp*, *luxCDABE*) chromosomal insert, also known as FSC1363	This study
FSC200	*holarctica* (type B)	Human ulcer, 1998, Sweden (77)
FSC200 *gfp lux*	FSC200 with (*gfp*, *luxCDABE*) chromosomal insert, also known as FSC1364	This study
LVS	*holarctica* (Live Vaccine strain), also known as FSC155	Live vaccine strain from Russia modified in USA (78)
*E. coli* strains		
Top10	F^–^ *mcrA* (*mrr-hsdRMS-mcrBC*), 80*lacZ*M15 *lacX74 recA1 deoR araD139* (*ara-leu*)*7697 galU galK rpsL* (Sm^r^) *endA1 nupG*	Invitrogen, USA
TransforMax EC100D *pir+*	*F - mcrA Δ(mrr-hsdRMS-mcrBC) φ80dlacZΔM15 ΔlacX74 recA1 endA1 araD139 Δ(ara*, *leu)7697 galU galK λ- rpsL (Str R) nupG pir +(DHFR*)	Lucigen, USA
S17-1λ*pir*	*recA*, *thi*, *pro*, *hsdR*^–^*M+*, <RP4:2-Tc:Mu:Km:Tn*7* > Tp^R^, Sm^R^	(79)
Plasmids		
pDM4	Suicide plasmid; SacB, mobRP4, oriR6K, Cm^R^	(80)
pDM4*igs*	pDM4 with 800 bp flanking sequences complementary to *igs*	This study
pXen-13	pSK *luxCDABE*, Amp^R^	Caliper Life Science, USA
pKK214-*gfp*	*groELS* promoter, Ft ori, p15A ori, *gfp*, Tc^R^	(41)
pKK289Kan-*gfp*	*groELS* promoter, Ft ori, p15A ori, *gfp*, Kan^R^	(81)
pKKB31	pKK289Kan-*gfp* containing a *bfr-gfp-luxCDABE* cassette	This study

### Fifty-four-week incubation axenic system setup

To investigate the impact of discrete environmental factors on long-term persistence of the virulent strains Schu S4 (type A) and FSC200 (type B; Fig. 1; Table 1), we set up closed axenic systems with eight different conditions. We monitored persistence by sampling cell populations at 0, 6, 12, 28, and 54 weeks for (i) culturable counts by growth on GC II solid agar after serial dilution in 0.9% NaCl, (ii) total cell counts by SYBR Green stain and flow cytometry, (iii) quantitative DNA analysis by real-time PCR targeting the *F. tularensis*-specific 17 kDa region (Table 4), and (iv) cell integrity assessment by real-time PCR targeting the 17 kDa region following treatment with PMA (Table 4). To verify persistence and the ability to perpetuate disease of non-culturable cells (i.e., to distinguish between dead intact cells from virulent non-culturable intact cells in the bacterial population incubated for 54 weeks), we used a mouse infection model. Incubation conditions (Table 1) were chosen to provide a test of the following factors: two different starting densities of *F. tularensis* cells (10^9^ CFU/mL and 10^4^ cells CFU/mL), three levels of salinity (0.9% NaCl, 3.6% NaCl, and 0% NaCl using sterile deionized water [Ultrapure water]), two temperatures (RT as a range from 19 to 22°C and 4°C), three levels of nutrient availability (oligotrophic, mesotrophic, and eutrophic [82]), and initial presence/absence of amoeba cells (*Acanthamoeba castellanii*). Baseline conditions for the original persistence model used are listed in the third row of Table 1 (10^9^ CFU/mL, 4°C, 0.9% NaCl, absence of *A. castellanii*, and oligotrophic nutrient level). The experiment was set up from bacteria harvested from GC II solid agar and suspended in NaCl at 10^9^ cells/mL, determined by optical density measurements at OD_600_ nm. The bacterial suspension was transferred to 1.5 mL Eppendorf tubes (1 mL in each) in three biological replicates, with three technical replicates per biological replicate. Tubes were incubated in the dark. The C:N:P molar ratio of nutrients added was 60:10:1, in accordance with the stoichiometry of bacteria (83). A higher nutrient condition was achieved by adding organic C (glucose) and inorganic N and P (ammonium chloride and sodium phosphate) to the NaCl solution. A culture of *A. castellanii* was produced by inoculation into ATC 217 PYG medium and incubated for 5 days at RT. Protozoa were enumerated by counts using a Bürkner chamber. The *A. castellanii* culture was spun down by centrifugation and resuspended in 0.9% NaCl. The 1-year incubations were inoculated to a final concentration of 10^4^ amoebae/mL.

**TABLE 4:** Primers used in the study

Primer/probe	Primer/probe sequence	Target	Source/reference
Ft-sp. FTS_0772-F	CAAGGTAAAGAAATTAAGAGTAGTAAAGTTGAATTC	*hsdS*, partial locus	(1)
Ft-sp. FTS_0772-R	ATTTAATCTAGTATTATCAATTGGGTAAAAAGGTA	*hsdS*, partial locus	(1)
TaqMan-MGB probe	FAM-GATGTGGCAACAACTGAAAT		(1)
17kD42-F	CGCAGGTTTAGCGAGCTGTT	*lpnA*	This study
17kD308-R	GGAAGCTTGTATCATGGCACTT	*lpnA*	This study
iQFt1-F	CGCAGGTTTAGCGAGCTGTT	*lpnA*	(38)
iQFt1-R	GCAGCTTGCTCAGTAGTAGCTGTCT	*lpnA*	(38)
Bact_341F	CCTACGGGNGGCWGCAG	V3V4 region of the bacterial 16S rRNA	(84)
Bact_785R	GACTACHVGGGTATCTAATCC	V3V4 region of the bacterial 16S rRNA	(84)
bfr5Nhe	ctagctagctcgagatccatacccatgatggttac	*brf*	This study
bfr3Pst	aatctgcagatcaataatttcttgtttattttc	*brf*	This study

### Eighty-five-week incubation axenic system setup

We set up closed axenic systems as described above for the 54-week study with the exception that incubation was prolonged to 85 weeks, only employing the baseline treatment conditions (10^9^ CFU/mL, 4°C, 0.9% NaCl, absence of *A. castellanii*, and oligotrophic nutrient level) and set up with the Schu S4 *gfp lux* and FSC200 *gfp lux* strains. The incubations were sampled at time points 0, 12, 21, 31, 35, 56, and 85 weeks. At each time point, viable counts (presented as CFUs/mL) of strains Schu S4 *gfp lux* and FSC200 *gfp lux* were analyzed. To test for significant differences in decline of CFUs between the two strains, the area under the curve values for each biological replicate were compared across the two strains using an unpaired *t*-test. *P* values < 0.05 were considered statistically significant. To verify persistence and the ability to perpetuate disease of non-culturable cells (i.e., to distinguish between dead intact cells from virulent non-culturable intact cells in the bacterial population incubated for 35, 56, and 85 weeks), we used a mouse infection model.

### Total counts

Samples from the axenic system setup were fixed with 2% glutaraldehyde and diluted to contain approximately 10^6^ cells/mL. The samples (495 µL) were stained with 5 µL SYBR Green I (Invitrogen AG, Switzerland; 1:100 dilution in DMSO, Sigma Aldrich) and incubated in the dark at 37°C for 15 min. After incubation, the samples were analyzed with flow cytometry according to protocol (85) using a BD Accuri C6 Plus instrument (Becton Dickinson, Belgium). Every sample was analyzed in triplicate with a flow rate of 35 µL/min and a threshold of 500 units. Events were detected with green fluorescence channel (FL-1, 533 ± 30 nm) and red fluorescence channel (FL-3, >670 nm), and data were processed with the FlowJo software (Tree Star Inc., USA) to determine total cell count.

### Real-time PCR and PMA PCR

The quantity of *F. tularensis*-specific genome copies and the proportion of freely available DNA due to cell damage were monitored by qPCR and a PMA PCR assay, respectively. From each sample, 50 µL was pipetted into two Eppendorf tubes, centrifuged for 10 min at 17,000 × *g*, whereupon the supernatants were removed. The pelleted bacteria were resuspended in 400 µL 10 mM Tris-EDTA. To one of the two tubes, 4 µL of 5 mM PMAxx (Biotium) was added, and all samples (with and without PMAxx) were incubated for 10 min on a Thermomixer comfort (Eppendorf) in the dark with constant agitation at 300 rpm. Thereafter, the samples were exposed to UV light for 15 min (PMA-Lite, Biotium), and the bacteria were pelleted by centrifugation for 10 min at 17,000 × *g*. The supernatant was removed, the pellet was resuspended in 200 µL water, and DNA was extracted with magLEAD 6gC (PSS BioSystem Net) according to the manufacturer’ instructions. Each sample was eluted in 200 µL and stored at −20°C until analysis. The CFX96 Touch Real-Time PCR detection system was used to quantify genomic DNA specific for *F. tularensis*. Each qPCR was performed in Twin.tec real-time PCR plate 96 (Eppendorf) that was sealed with an optical adhesive cover (VWR). PCR reactions were performed in a final volume of 25 μL containing 1× Ssofast Evagreen supermix (BioRad), 400 nM of 17kD42f- primer, 400 nM of 17kD308r- primer (Table 4), and 1 μL of DNA extracted with or without use of PMA treatment. Cycling conditions used in this study were 2 min at 98°C, followed by 40 cycles of 5 s at 98°C and 5 s at 58°C, ending with a melt curve (70°C–95°C, 0.5°C/s). In all cases, a negative amplification control was included using 1 μL of water instead of genomic DNA. The signal reduction effect of PMA was expressed as “damaged bacteria” obtained by subtraction of quantification Ct values of non-PMA-treated cell suspensions from the Ct values of the corresponding PMA-treated cell suspensions (Table 1).

### Animal challenge

Mice were infected to test for persistence and virulence in three separate challenges: (i) Schu S4 and FSC200 cell populations for the 54-week axenic system (Fig. 1; Fig. S2), (ii) the Schu S4 *gfp lux* and FSC200 *gfp lux* populations for the 85-week experiment and dilution series (Fig. 2A and 3), and (iii) the Schu S4 *gfp lux* and FSC200 *gfp lux* populations after 10 weeks in the pond water study setup (Fig. 5; Fig. S2). In all experiments, C57BL/6J female mice, aged 7–10 weeks (Scanbur AB, Sollentuna, Sweden), were used for virulence studies. Mice were injected intraperitoneally with 100 or 200 µL of the *F. tularensis* incubations. For positive control infections, *F. tularensis* strains were grown for 16 h on GC II solid agar plates before the bacteria were suspended in phosphate-buffered saline (PBS) pH 7.4 to an OD_540_ = 0.35, which normally corresponds to approximately 10^9^ CFU/mL. The bacterial suspension was diluted in PBS or NaCl into two doses used for challenge, around 10 and 100 bacteria for the Schu S4 or Schu S4 *gfp lux* strain, and 100 and 1,000 bacteria for the FSC200 or FSC200 *gfp lux* strain, in a total volume of 100 μL. The infection doses were determined by viable count analysis. For the serial dilution of the 85-week sample of non-culturable FSC200 *gfp lux* populations, three steps of 100× dilutions (i.e., 10^2^×, 10^4^×, and 10^6^×) were performed with 0.9% NaCl. Dilutions were used as inocula for mouse challenges. All experimental samples were administered in replicates of three to seven mice per sample. As a positive control, at least two mice were injected via the IP route with *F. tularensis* cells grown on GC II solid agar as described above. For negative controls, at least two mice were injected with 100 µL of PBS, 0.9% NaCl, or filtered pond water. Mice were observed for weight drop that precedes the visible symptoms of murine tularemia (i.e., > 1.2 g of weight drop within a single 24-h period). Moribund mice were euthanized immediately, and all remaining mice were euthanized after 20 days. All virulence tests in mice were performed under BSL-3 conditions. The presence of *F. tularensis* in euthanized mice was confirmed by serial dilutions of homogenized spleens plated on GC II agar plates as described above and confirmatory real-time PCR using the iQFt1 primer pair (Table 4). The Kaplan-Meier estimator was used to estimate survival proportions and statistical analysis with log-rank test for comparison of survival functions between groups (GraphPad Prism software). *P* values < 0.05 were considered statistically significant.

### Evaluation of construct strains—growth, intracellular survival, and luminescence

A chromosomal construct in *F. tularensis* that constitutively expresses the *Photorhabdus luminescens* lux operon (*luxCDABE*) and green fluorescent protein (GFP) was developed and introduced into strains Schu S4 and FSC200. Several intergenic sequences (*igs*) with a length of greater than 1 kb were identified, and a 1,111 bp region, located between the *rho* and *manB* genes, was selected for integration of the construct. ManB is involved in O-chain biosynthesis in *Francisella*, and *manB* mutants have a distinct phenotype allowing for detection of downstream polar effects (86). Using the SacB-based pDM4 vector (80), an *igs*-vector containing an integration cassette with 800 bp flanking sequences complementary to the sequences on the intergenic sequence was developed. The integration cassette, containing the sites for *Sal*I and *Sac*I restriction enzymes at the 5′ and 3′ ends of the flanking regions as well as several other restriction sites, was commercially synthesized by GenScript and cloned into the pDM4 vector, resulting in a pDM4*igs* vector (this study). In *F. tularensis*, the natural bacterioferritin (*bfr*) promoter drives the expression of bacterioferritin, an iron-storage protein. While iron typically regulates ferritin-family genes in many bacteria, the native *F. tularensis bfr* promoter is generally utilized in molecular genetics as a strong, constitutively active housekeeping promoter rather than an inducible switch. A side-by-side quantitative comparison of GFP expression driven by the *bfr* and the *groEL* promoter suggests that the *bfr* promoter is about 10-fold more potent than the *groEL* promoter (87). The *bfr* promoter (87) from the live vaccine strain was amplified by PCR and cloned into the pKK214-*gfp* vector (41), resulting in pKK214-*bfr-gfp*. The *luxCDABE* operon was excised from the commercial pXen-13 plasmid (Caliper Life Science, U.S.) by *Eco*RI/*Sac*I digestion and cloned into the *Eco*RI/*Acl*I digested pKK289Kan-*gfp* plasmid (81), resulting in a pKK289Kan-*gfp-luxCDABE* plasmid. Next, the *bfr-gfp* region from pKK214-*bfr-gfp* was excised by *Nhe*I/*Sac*I digestion and cloned into the pKK289Kan-*gfp-luxCDABE* plasmid, substituting *gro-gfp* for the *bfr-gfp* sequence. The resulting plasmid, designated pKKB31, had both the *gfp* and *luxCDABE* under control of the *bfr*-promoter (this study). The *bfr-gfp-luxCDABE* cassette from the pKKB31 plasmid was finally cloned into the suicide vector pDM4*ig*s and inserted into the chromosome of the Schu S4 strain and the FSC200 strain by allelic replacement (88). Resulting strains, designated Schu S4 *gfp lux* and FSC200 *gfp lux* (Table 3), contained the *bfr-gfp-luxCDABE* construct inserted at 968 bp downstream of *rho* and 143 bp downstream of the *manB* gene. We verified that the constructs, Schu S4 *gfp lux* and FSC200 *gfp lux*, behaved as their parental strains, Schu S4 and FSC200, with regard to virulence (Fig. S2), growth rate in Chamberlain’s growth media, intracellular infection of macrophages (J774), and persistence in co-incubation with amoeba (10^4^ *A. castellanii*) in 0.9% NaCl at 4°C (Fig. S3).

Furthermore, the activity of *F. tularensis* construct strains was evaluated by measuring luminescence (Tecan Infinite F Plex; Fig. S5). Bacterial strains were grown for 16 h on GC II solid agar plates before the bacteria were suspended in PBS pH 7.4 to an OD_540_ = 0.35, which normally corresponds to approximately 10^9^ CFU/mL. The bacterial suspension was diluted in PBS for starting inocula in a range from 2 × 10^6^ bacteria down to two bacteria. A total of 20 µL of bacterial dilution was added to 180 µL Chamberlain medium in a black plate with a clear bottom (ThermoFisher Scientific, USA). The software Magellan was used to create an experiment setup with incubation at 37°C with shaking. Luminescence was measured every second hour for 20 cycles (a total of 40 h).

### Natural pond water study setup

To mimic natural water, we used pond freshwater to investigate the effect of a complex microbial community on the persistence of strain Schu S4 *gfp lux* and strain FSC200 *gfp lux* (Table 2). The pond water experiment introduced microbial complexity and utilized pond freshwater instead of 0.9% NaCl used in the 54- and 85-week axenic incubation studies. With this experimental model, we addressed whether the *F. tularensis* populations can persist in a complex environment with an active microbial community. We also investigated the possibility that *F. tularensis* persistence may be favored by low temperature, as observed in the 1-year axenic setup (RT compared to 4°C; Table 1). Pond water was collected in August (water temperature 17°C) from a beaver pond in Bäckmyrbäcken (64°03′27.6″ N 20°19′49.3″ E), northern Sweden, prefiltered through a 90 µm nylon mesh to exclude debris, and stored in acid-rinsed ten L glass bottles. In the laboratory, two pond water treatments, with and without microbial complexity, were set up by using the eluate after filtering natural pond water with different-sized filters: water sterilized using a 0.2 μm filter and water plus bacteria and protozoans using a 200 μm filter (Table 2). The pond water (200 µL from each filter treatment) was confirmed negative for naturally occurring *F. tularensis* DNA by real-time PCR targeting a *F. tularensis*-specific region (using the Ft-sp. FTS_0772 primers and probe, Table 4). Actively growing strains SchuS4 *gfp lux* and FSC200 *gfp lux* were inoculated into each treatment to a final concentration of 1.2 × 10^8^ and 2.6 × 10^8^ CFU/mL, respectively, with three biological replicates per treatment. The entire design at 100 mL in each container was replicated at two temperatures (RT and 4°C). To simulate natural water, an excess of pond water was filtered using a 0.2 μm filter. This filtered pond water was stored at 4°C, and every second day, it was used for continuous replacement of 10% of the volume of the containers. The pond water was characterized at the time of collection (SGS Analytics Sweden AB): pH 4.5, conductivity 3.8 mS/m, TOC 40 mg/L, Tot-N 0.82 mg/L, Tot-P 0.022 mg/L, Fe 2.1 mg/L, Ca 2.3 mg/L, K 0.24 mg/L, Mg 0.54 mg/L, Mn 0.020 mg/L, and Na 1.3 mg/L. We monitored the persistence of the *F. tularensis* populations by sampling at 0, 1, 2, 4, 6, and 8 weeks for: culturable counts by growth on GC II solid agar, quantification of DNA by real-time PCR targeting a *F. tularensis*-specific region (using the Ft-sp. FTS_0772 primers and probe, Table 4), composition of the bacterial communities (amplicon sequencing of a 16S rRNA gene fragment, Table 4), and measurement of luciferase activity upon addition of nutrients. To verify the persistence of *F. tularensis* incubated for 10 weeks, we used a mouse infection model as described above. Measurement of relative luminescence (Lux) of Schu S4 *gfp lux* and FSC200 *gfp lux* from pond water samples after inoculation into rich growth media (Chamberlain defined medium) and incubated at 37°C. Relative Lux was measured every hour for 38 h (Tecan Infinite F Plex). A total of 20 µL of each pond water incubation sample was added to 180 µL of Chamberlain defined medium in a black plate with a clear bottom (ThermoFisher Scientific, USA). The software Magellan was used to create an experiment setup with incubation at 37°C with shaking. OD_600_ and luminescence were measured every second hour for 20 cycles (a total of 40 h).

### *Francisella*-specific real-time PCR from pond water

A total of 200 µL of each sample was subjected to DNA extraction with the MagDEA Dx SV kit on the magLEAD 6gC (PSS bio system net), according to the manufacturers instructions. Each sample was eluted in 200 µL and stored at −20°C until analysis. Quantification of *F. tularensis*-specific DNA was performed by real-time PCR using primers and probes, Ft-sp. FTS_0772 primers and TaqMan-MGB probe (Table 4). Quantification of *Francisella*-specific DNA was carried out by using a BioRad CFX96 Real-Time System (BioRad) with SsoAdvanced Universal probemix (BioRad). For PCR amplifications, the reactions were performed in a total volume of 20 µL, containing 1 µL of the DNA sample, 100 nM of each of the primers, 100 nM of the fluorogenic probe, respectively, and 16 µg Bovine Serum Albumin (Roche, Germany). The PCR condition for the *F. tularensis*-specific DNA amplification started with a DNA denaturation step at 95°C for 3 min, followed by 45 cycles of denaturation at 95°C for 3 s, followed by annealing and synthesis at 60°C for 30 s. *F. tularensis*-specific genome equivalents (GE/mL) were estimated from the qPCR values using a linear model fitted to the calibration curve consisting of six points ranging from 3.3 × 10^3^ to 3.3 × 10^8^ CFU/mL. The real-time PCR-derived genome equivalents were assigned a floor value of 10³ GE/mL for samples below the limit of detection for the real-time PCR and log10 transformed. Reduction of *F. tularensis*-specific GE was analyzed using ANCOVA with time as a continuous predictor and strain, incubation temperature, and pond water filter size as categorical predictors. Interaction terms between time and the categorical predictors were used to test whether the slopes of GE reduction differed among conditions. *P* values < 0.05 were considered statistically significant. The calculations were conducted in R (R Core Team 2025) using the tidyverse library (89).

### Amplicon sequencing library preparation from pond water

The V3V4 region of the bacterial 16S rRNA gene was amplified using universal bacterial primers Bact_341F and Bact_785R (Table 4), including golay barcode sequences. Extracted samples from week 0, 1, 2, 4, 6, and 8 in the pond water study were amplified in triplicate. Each PCR reaction contained 12.2 µL of nuclease-free water, 10 µL of 2.5 × 5 PRIME HotMasterMix (Quantabio, USA), 0.8 µL of 10 mg/mL Bovine Serum Albumin (Roche, Switzerland), 0.5 µL of forward and reverse primer, respectively, to a final concentration of 10 µM, and 1 µL DNA template. The PCR was performed under the following conditions: initial denaturation at 94°C for 3 min, followed by 35 cycles of 94°C for 45 s, 50°C for 1 min, and 72°C for 1 min, with the final extension step at 72°C for 10 min. The amplified triplicates were pooled before being evaluated with agarose gel electrophoresis and quantified with the Qubit 2.0 dsDNA HS Assay Kit (Thermo Fisher Scientific, USA). A total of 50 ng of each replicate was pooled together, resulting in a sequencing library. The library was purified with QIAquick PCR Purification Kit (Qiagen, Germany), denatured, and diluted according to the manufacturer’s instructions (Illumina, USA). Final concentration of the sequencing library was 10 pM with 5% PhiX. Sequences were obtained on the Illumina MiSeq platform using a MiSeq Reagent Kit v3 (600 cycles; Illumina, USA).

### Amplicon sequence analysis

Illumina sequences were preprocessed and annotated with DADA2 (v 1.24.0 [90]) pipeline (1.8) using default parameters and Silva v138.1 taxonomy database. Analysis of the read quality, however, resulted in discarding reverse reads due to insufficient quality, and taxonomic assignments are based on forward reads only. Annotated samples were analyzed and visualized using R packages Phyloseq (v 1.42.0 [91]), Phylosmith (v 1.0.6 [92]), and MicroViz (v0.10.8 [93]).

## Data Availability

Raw whole-genome sequencing reads from construct strains and pond water 16S rRNA gene amplicon sequencing reads have been deposited in the NCBI Sequence Read Archive (SRA) under BioProject accession PRJNA1481474.

## SUPPLEMENTAL MATERIAL

Supplemental material aem.01261-26-s0001.docx Fig. S1 to S6.

ASM does not own the copyrights to Supplemental Material that may be linked to, or accessed through, an article. The authors have granted ASM a non-exclusive, world-wide license to publish the Supplemental Material files. Please contact the corresponding author directly for reuse.
