# Supplementary material for: A slow decay to extinction: cold temperatures and reduced microbial complexity prolong the persistence of planktonic Francisella tularensis
Source: Appl Environ Microbiol. 2026 Sep 29;92(10):e01261-26. doi: 10.1128/aem.01261-26 (PMC13689442; doi:10.1128/aem.01261-26)
Supplement: Supplementary file 1 [file aem.01261-26-s0001.docx]

**Supplementary Figure 1.** Long-term persistence of wild type *F. tularensis* Schu S4 (Type A) and FSC200 (Type B) strains in axenic conditions incubated at different temperature (4°C and RT), different NaCl concentrations (0, 0.9 and 3.6%), varying nutrient availability (mesotrophic and eutrophic) and presence of amoeba (*A. castellanii*). In this experiment most cultures were incubated at 4°C and the varying conditions were chosen to evaluate the potential for long-term persistence in cold water. Bars on each graph represent data from sampling the seven different culture conditions (color coded) at five time points: 0, 6, 12, 28 and 54 weeks; absent bars represent measurements below the limit of detection. Error bars are from three biological replicate cultures. The dashed line in the graph represents the limit of detection for the analysis. **A.** Total bacterial counts from flow cytometry measurement of *F. tularensis* cells stained with SYBR Green. **B.** Results of quantitative real-time PCR assay for all available *F. tularensis* DNA, which included both undamaged and damaged cells. A lower Ct value indicates higher amounts of DNA, which is why the y-axis is reversed on this graph. **C.** Proportion of damaged cells measured using PMA PCR assay. Greater delta-Ct values indicate a greater number of damaged *F. tularensis* cells in the sample. Samples with lower starting inocula (10^4^ cfu/mL) were not included in the graph since the concentration was below the detection rate for flow cytometry analysis and PCR at the 6 week sampling point.

**Supplementary Figure 2.** Survival proportion of positive and negative control mice. Positive control mice infected with ten-fold dilutions of actively growing Schu S4 (Type A) and FSC200 (Type B) populations (range from 100 down to 1 bacteria and 1000 down to 10 bacteria, respectively) and construct strains Schu S4 *gfp lux* and FSC200 *gfp lux* (infectious dose 100 bacteria). Negative control mice received saline solution (0.9% NaCl), and pond water filtered at 200 µm filter size and 0.2 µm filter size. Data are presented as survival curves of mice (n ≥3) after i.p. infection at day 0. Each non-negative event represents a mouse confirmed with tularemia. The observed difference in survival times between the two wild type strains (Schu S4 and FSC200) were significantly different by Log rank test. An earlier onset of symptoms occurred in mice infected with the same infectious dose (10^1^ cfu and 10^2^ cfu) of Schu S4 compared to FSC200 (*p* <0.05). Survival curves for construct strains Schu S4 *gfp lux* and FSC200 *gfp lux* were not statistically different from parental strains at the same infectious dose (10^2^ cfu). Note the scale brake of the x axis.

**Supplementary Figure 3.** Comparison of wild type parental strains Schu S4 and FSC200 and construct strains Schu S4 *gfp lux* and FSC200 *gfp lux.* **Panel A.** Bacterial growth in Chamberlain’s growth media at 37°C. **Panel B.** Infection of J774 cells at 37°C. **Panel C.** Co-incubation with 10^4^ *A. castellanii* cells in 0.9% NaCl at 4°C.

**Supplementary Figure 4.** Results from pond water maintained at 4°C (**Panel A**) or RT (**Panel B**) filtered through a 0.2 um filter (upper panels) or a 200 μm filter (lower panels), inoculated with Schu S4 *gfp lux* (left panels) and FSC200 *gfp lux* (right panels) and measured at 0, 1, 2, 4, 6, and 8 weeks (x-axis). Relative abundance (left y-axis) of major bacterial taxa (legend) was determined via 16S rRNA amplicon sequencing, and *F. tularensis* specific genome equivalents (right y-axis) per mL pond water were inferred from a *F. tularensis*-specific TaqMan PCR assay. Bars with no standard deviation represent samples with *F. tularensis* below the limit of detection for the PCR assay (< 10^3^ bacteria/mL). Taxa of relative abundance <5% were categorized as Other. Bacterial populations that were still virulent for mice at 10 weeks are marked with*.

**Supplementary Figure 3.** Relative abundance (left y-axis; determined via 16S rRNA amplicon sequencing) of major bacterial taxa (legend) and. Taxa of relative abundance <5% were categorized as Other.

**Supplementary Figure 5.** The relative luminescence (Lux) emitted by the construct strains *F. tularensis* Schu S4 *gfp lux* (**left panel**) and FSC200 *gfp lux* (**right panel**) during active growth in Chamberlain’s liquid medium at 37°C. Growth curves were generated from a dilution series of starting inocula that ranged from 2 x 10^8^ bacteria down to ten bacteria. Relative luminescence was measured every second hour for 40 hours using a Tecan Infinite F Plex reader and Magellan software.

**Supplementary Figure 6**. Measurement of relative luminescence (Lux) of Schu S4 *gfp lux* (**left panel**) and FSC200 *gfp lux* (**right panel**) in 0.2µm (control) and 200µm (complex microbial background) filtrate pond water background after inoculation into rich growth media (Chamberlain) at 37°C. The four different pond water samples were withdrawn from the pond water setup minutes after initiation (time 0). These experiments utilized inocula only from the RT microcosms, so no temperature effects were studied. Realtive Lux was measured every second hour for 38h. The 20 µl inocula of pond water used to initiate these experiments contained approximately 2.4 x 10^6^ or 5.2 x 10^6^ CFUs of Schu S4 *gfp lux* or FSC200 *gfp lux*, respectively.
